# Supplementary material for: Diet modifies the association between alcohol consumption and severe alcohol-related liver disease incidence
Source: Nat Commun. 2024 Aug 11;15:6880. doi: 10.1038/s41467-024-51314-9 (PMC11317484; doi:10.1038/s41467-024-51314-9)
Supplement: Supplementary file 1 — Supplementary Information [file 41467_2024_51314_MOESM1_ESM.pdf]

Supplementary Material

Diet modifies the association between alcohol and severe alcohol-related liver disease: findings from UK Biobank

Supplementary Figure 1. Coefficients of included dietary items in models with different lambda values (L1 Norm)........ 2

Supplementary Figure 2. Correlation matrix among the 20 dietary items 3

Supplementary Figure 3. Cross-validated C-index of the optimal λ values 4

Supplementary Table 1. Pathologies and drug use disorder at/before baseline excluded from the analyses. 5

Supplementary Table 2. Individual association between dietary items and severe ALD. 6

Supplementary Table 3. Coefficients of the LASSO model at the chosen lambda........ 7

Supplementary Table 4. Interaction between per 7-units/week of alcohol intake (continuous variable) and diet score with severe ALD 8

Supplementary Table 5. Interaction between alcohol intake and diet score with specific ALD outcomes 9

Supplementary Table 6. Population attributable fraction of ALD diet score and units/week of alcohol consumption (continuous variable)........ 10

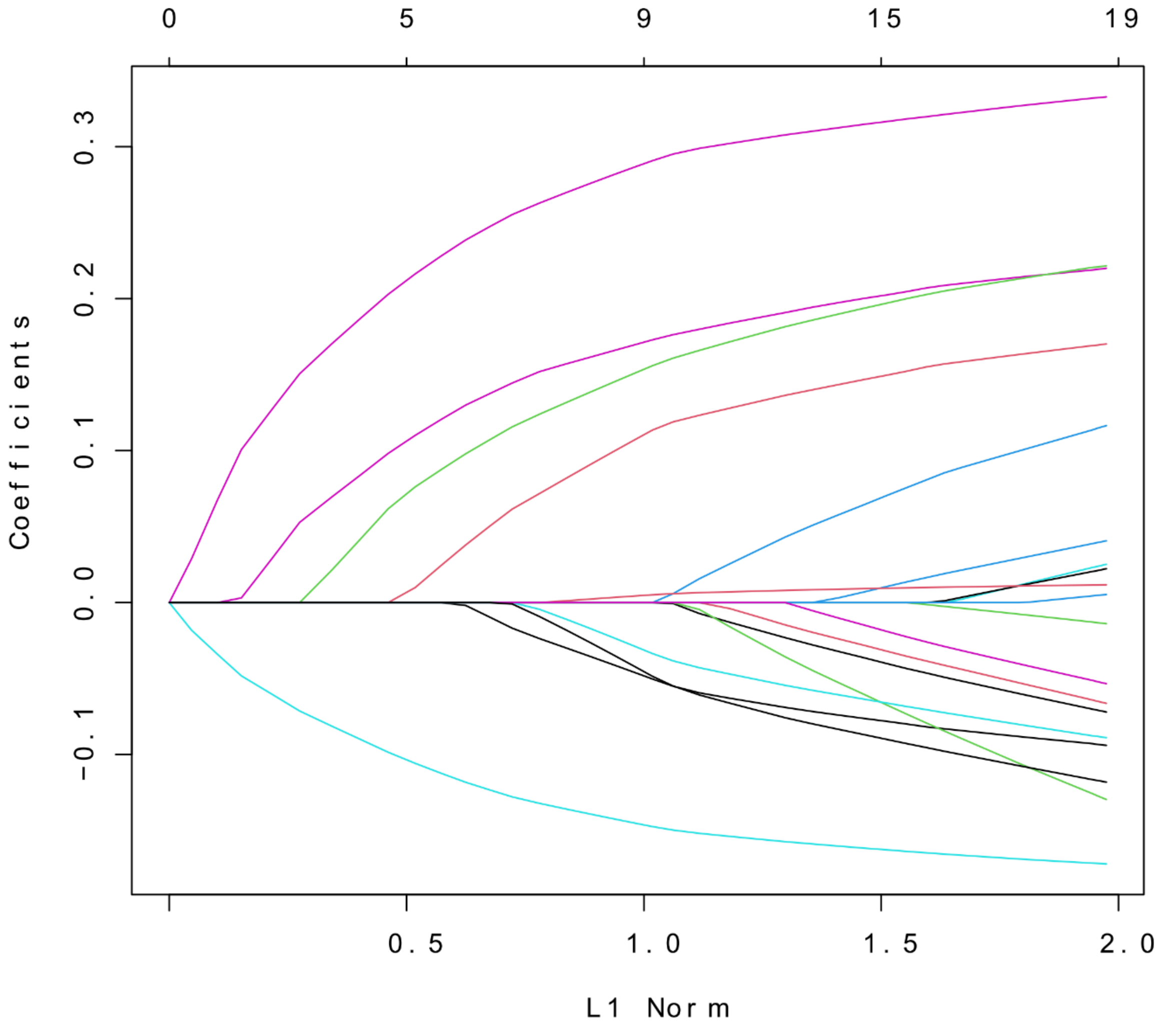

Supplementary Figure 1. Coefficients of included dietary items in models with different lambda values (L1 Norm).

Coloured lines shown are the reduction in the dietary items' reduction in coefficient as L1 norm is reduced. Purple, cyan, magenta, green, orange, and two black lines showed the coefficients of salt added to food, cereal, processed meat, beef, lamb, coffee and tea intake. Analyses were estimated by Cox regression models using the LASSO algorithm.

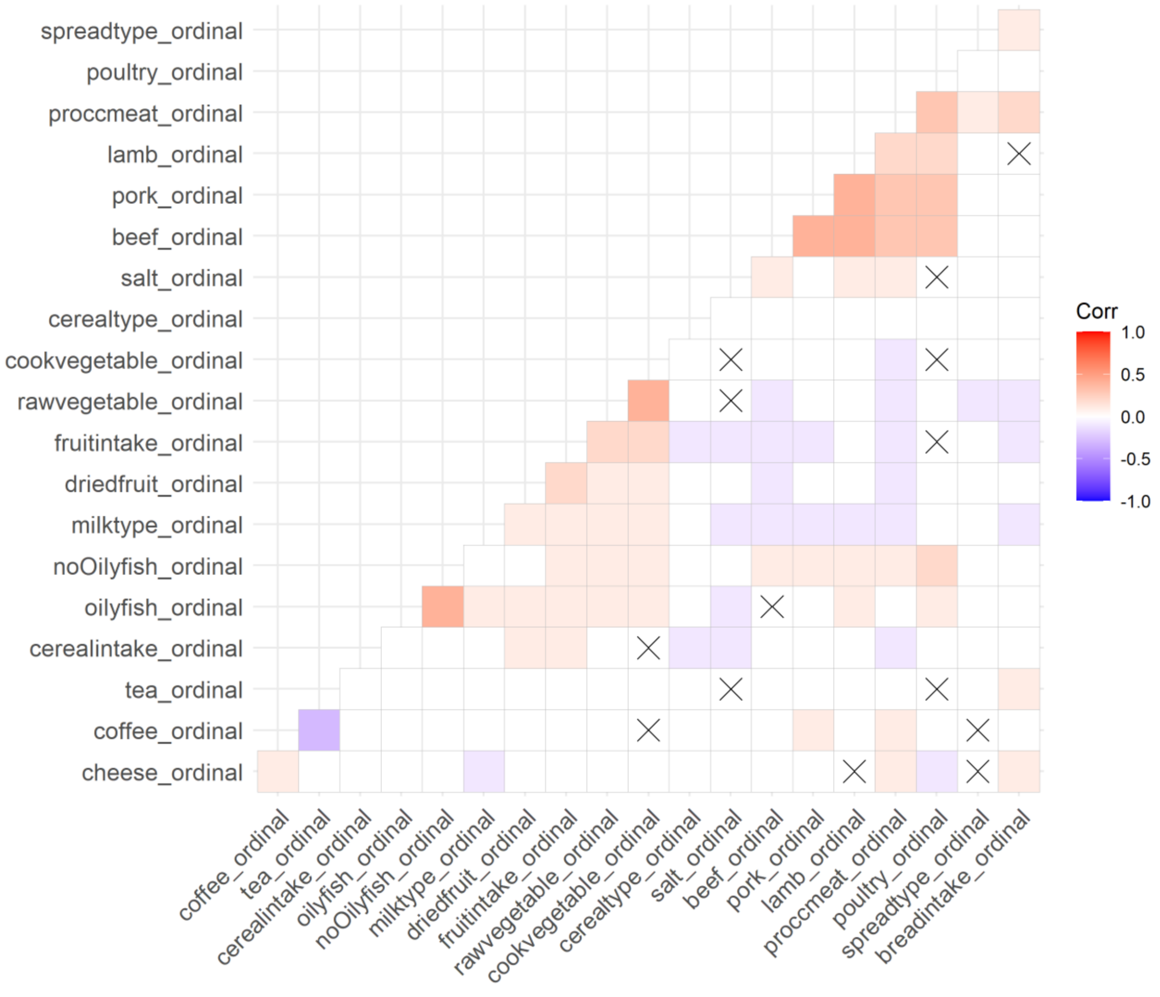

Supplementary Figure 2. Correlation matrix among the 20 dietary items
The coloured showed are the Spearman's correlation coefficients. Xs represent non-significant correlation. Analyses were estimated by Cox regression models using the LASSO algorithm.

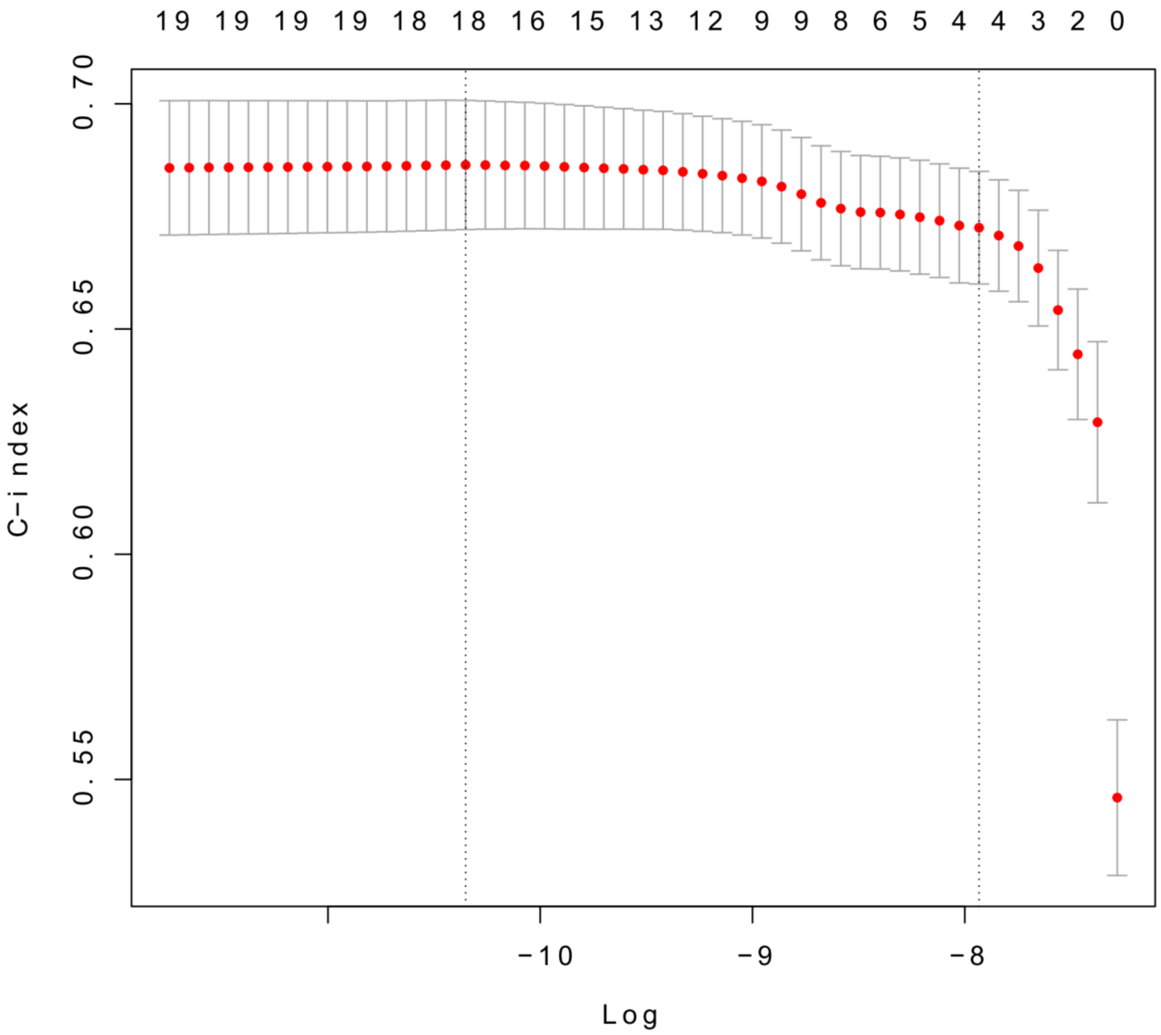

Supplementary Figure 3. Cross-validated C-index of the optimal λ values
Numbers on top showed the number of dietary items being selected for different lambda values. The two dotted lines showed the maximal (left) and the within 1-SD C indices. This validation aimed to maximise Harrel´s C-index, where a higher C-index indicates superior risk discrimination performance. Analyses were estimated by Cox regression models using the LASSO algorithm.

Supplementary Table 1. Pathologies and drug use disorder at/before baseline excluded from the analyses.

**Diagnosis**	**ICD-10**
**Excluding at baseline**	
ALD	K70 (all: from 70.0 to 70.9)
Viral Hepatitis	B16, B17, B18, B19
Autoimmune liver disease (AIH, PBC, PSC)	K83.0A, K83.0F, K74.3, K75.4
Hemochromatosis	E83.1
Wilson	E.83.0B
Alpha-1-antirypsin deficiency	E88.0A, E88.0B
Budd-Chiari	I82.0, K76.5
Chronic hepatitis, unspecified	K73.9, K73.2
Secondary or unspecified biliary cirrhosis	K74.4, K74.5
NAFLD/NASH	K76.0 & K75.8
**Excluding drug use disorders at/before baseline**	
Codes associated with drug use disorders except nicotine/caffeine	F11-F14, F16, F18, F19

Supplementary Table 2. Individual association between dietary items and severe ALD.

Dietary item	Unit	HR (95% CI)	p-value
Cooked vegetable intake	Portions/day	0.96 (0.87; 1.06)	0.401
Salad/raw vegetable intake	Portions/day	1.04 (0.96; 1.12)	0.354
Fresh fruit intake	Portions/day	0.94 (0.89; 1.00)	0.065
Dried fruit intake	Portions/day	0.94 (0.87; 1.01)	0.086
Oily fish intake	Frequency/week	1.01 (0.92; 1.11)	0.804
Non-oily fish intake	Frequency/week	1.03 (0.92; 1.14)	0.611
Processed meat intake	Frequency/week	1.10 (1.01; 1.19)	0.003
Poultry intake	Frequency/week	0.97 (0.88; 1.07)	0.564
Beef intake	Frequency/week	1.14 (1.04; 1.26)	0.010
Lamb intake	Frequency/week	1.12 (1.00; 1.26)	0.044
Pork intake	Frequency/week	1.05 (0.94; 1.18)	0.357
Cheese intake	Frequency/week	0.95 (0.88; 1.03)	0.257
Milk type	Full cream, semi-skimmed, skimmed, alternative milk, never/rarely have milk	1.04 (0.96; 1.13)	0.315
Spread type	Butter, others, rarely use spread	0.98 (0.91; 1.06)	0.567
Bread type	White, brown, wholemeal, others	0.99 (0.98; 1.00)	0.033
Cereal intake	Bowls/week	0.88 (0.86; 0.91)	<0.001
Cereal type	Bran, biscuit, oat, muesli, others	1.03 (0.96; 1.11)	0.375
Salt added to food	Always, usually, sometimes, rarely/never	1.25 (1.15; 1.36)	<0.001
Tea intake	Cups/day	0.95 (0.92; 0.98)	0.001
Coffee intake	Cups/day	0.91 (0.87; 0.95)	<0.001

Data are presented as hazard ratio and their 95% confidence intervals (95% CI). All analyses were performed excluding people who developed the disease during the first two years of follow-up. Individual analyses were adjusted for age, sex, deprivation, ethnicity, the five components of the metabolic syndrome, alcohol intake at baseline, smoking and physical activity. Analyses were estimated using Cox proportional hazard models. There was no comparison among dietary items.

Supplementary Table 3. Coefficients of the LASSO model at the chosen lambda.

**Dietary items**	**Coefficient**
Processed meat intake	0.098
Beef intake	0.061
Cereal intake	-0.098
Salt added to food	0.202

Estimated from the LASSO model selected from Supplementary Figure 3. There is small correlation between the included food items (Supplementary Figure 2) which might have affected the stability of the coefficients.

Supplementary Table 4. Interaction between per 7-units/week of alcohol intake (continuous variable) and diet score with severe ALD

	HR (95% CI)	RERI (95% CI)	Multiplicative interaction (95% CI)
**Overall**		0.19 (0.08-0.31)	0.94 (0.90-0.97)
Lower ALD diet score (< median)			
7 units lower alcohol intake	1 (Reference)		
7 units higher alcohol intake	2.23 (1.65-3.01)		
Higher ALD diet score (≥ median)			
7 units lower alcohol intake	1.31 (1.26-1.36)		
7 units higher alcohol intake	2.74 (2.04-3.67)		

Data are presented as hazard ratio and their 95% confidence intervals (95% CI). All analyses were performed excluding people who developed the disease during the first two years of follow-up. Analyses were adjusted for age, sex, deprivation, ethnicity, the five components of the metabolic syndrome, alcohol intake at baseline, smoking and physical activity, and alcohol drinking frequency. RERI: relative risk due to interaction. Calculation of RERI based on Cox proportional model requires comparison between two points of the continuous variable. In this analysis the two points chosen were by 7-unit difference in alcohol intake.

Supplementary Table 5. Interaction between alcohol intake and diet score with specific ALD outcomes

	HR (95% CI)	RERI (95% CI)	Multiplicative interaction (95% CI)
**Alcohol-related cirrhosis**		2.34 (0.54-4.14)	1.68 (0.92-3.09)
Lower ALD diet score (< median)			
Lower alcohol risk	1 (Reference)		
Increasing alcohol risk	1.86 (0.85-4.07)		
Higher alcohol risk	10.42 (4.72-22.98)		
Higher ALD diet score (≥ median)			
Lower alcohol risk	1.09 (0.64-1.86)		
Increasing alcohol risk	4.30 (2.15-8.61)		
Higher alcohol risk	10.87 (5.15-22.96)		
**Alcohol-related hepatitis**		1.14 (-6.26, 8.53)	0.27 (0.05-1.33)
Lower ALD diet score (< median)			
Lower alcohol risk	1 (Reference)		
Increasing alcohol risk	7.07 (1.60-31.12)		
Higher alcohol risk	26.52 (4.42-159.08)		
Higher ALD diet score (≥ median)			
Lower alcohol risk	1.50 (0.18-12.43)		
Increasing alcohol risk	8.39 (1.57-44.83)		
Higher alcohol risk	33.14 (5.26-208.96)		

Data are presented as hazard ratio and their 95% confidence intervals (95% CI). All analyses were performed excluding people who developed the disease during the first two years of follow-up. Analyses were adjusted for age, sex, deprivation, ethnicity, the five components of the metabolic syndrome, alcohol intake at baseline, smoking and physical activity, and alcohol drinking frequency. RERI: relative risk due to interaction. Analyses were estimated using Cox proportional hazard models.

Supplementary Table 6. Population attributable fraction of ALD diet score and units/week of alcohol consumption (continuous variable)

	**HR (95% CI)**	**Population attributable fraction, % (95% CI)**
**ALD diet score**	1.55 (1.25-1.92)	28.7 (15.7-39.7)
**Alcohol consumption (per 7 units/week)**	1.23 (1.20-1.27)	55.3 (50.6-59.5)

Population attributable fractions assume the HR shown to be causal which could not be shown in this study. All analyses were performed excluding people who developed the disease during the first two years of follow-up. Analyses were mutually adjusted, and adjusted for age, sex, deprivation, ethnicity, the five components of the metabolic syndrome, frequency of alcohol consumption, smoking and physical activity. Analyses were estimated using Cox proportional hazard models.
